# Adaptive Potential of Intracolonial Genetic Variability in Coral Populations

**DOI:** 10.1002/ece3.72352

**Published:** 2025-11-05

**Authors:** Lutfi Afiq‐Rosli, Carlos M. Duarte

**Affiliations:** ^1^ Marine Science Program, Biological and Environmental Science and Engineering Division King Abdullah University of Science and Technology (KAUST) Thuwal Saudi Arabia

## Abstract

Coral reef recovery and growth largely depend on clonal propagation, yet the critical role of intracolonial genetic variability (IGV) is often underestimated in current research. This review highlights the gap between coral research and studies on ecosystems like seagrass meadows, where clonal dynamics and IGV are more thoroughly examined. We found that only a small fraction of coral studies address this essential aspect, leading to an underestimation of the coral populations' adaptive potential in response to environmental stressors. We explore methodologies for detecting clones in corals and discuss key concepts such as IGV, somatic mutations, polyploidy, and chimerism and their implications for the adaptive potential of coral reefs. Additionally, we propose a framework for estimating the potential number of adaptive genotypes, considering factors like growth rates and polyp density. We recommend that future coral genetics and genomics research incorporate these clonal concepts to accurately assess the adaptive potential of coral reefs.

## Graphical abstract

Intracolonial genetic variability (IGV) is key to coral reef resilience but remains underexplored in current coral research. This review compares corals with well‐studied clonal systems like seagrass meadows, discusses methods to detect IGV and estimate the number of potential new genotypes, and advocates incorporating these clonal concepts into coral genetics research to better capture reefs' adaptive potential.

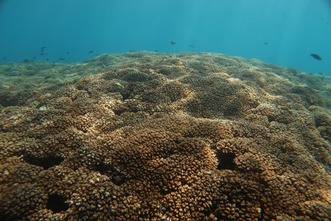

## 1 Introduction

Tropical coral reefs are a hyper‐diverse ecosystem that offers a plethora of services supporting close to an estimated million species (Fisher et al. 2015) and a billion people globally (Wong et al. 2022; Spalding et al. 2017; Beck et al. 2018). Unfortunately, coral reefs are undergoing an unparalleled loss because of cumulative impacts from anthropogenic disturbances, including deteriorated water quality, destructive fishing practices, and climate change, among others (Roberts 1995; Hobbs et al. 2015; Ballesteros et al. 2018; Cybulski et al. 2020). Severe heatwaves due to anthropogenic global warming, in particular, trigger episodes of mass bleaching, disrupting the symbiotic relationship between corals and their photosynthetic symbionts, often resulting in coral mortality (Hughes et al. 2017; Rädecker et al. 2021). The increased frequency and magnitude of heat waves with climate change leading to global mass bleaching events threaten the functional integrity of coral reef ecosystems by causing large projected global coral mortality (Hoegh‐Guldberg et al. 2018; Klein et al. 2022, 2024).

The persistence of coral reef ecosystems under such environmental pressure hinges on their capacity for adaptation and recovery (Drury 2020). Population connectivity and genetic diversity are central to this adaptive capacity, influencing the resilience and evolutionary potential of coral populations (Matz et al. 2018; Quigley et al. 2019). Although substantial research has focused on genetic diversity and population connectivity in corals, one critical aspect contributing to coral genetic diversity and, thus, fitness—intracolonial genetic variability (IGV)—remains largely overlooked (Oury and Magalon 2024). Clonal growth is the main mechanism allowing for the rapid recovery of corals following disturbance (Kayal et al. 2018), as exemplified by the rapid reported recovery of coral cover in the Great Barrier Reef in just 2–5 years following the 2016/2017 mass bleaching event (Morais et al. 2023; Australian Institute of Marine Science 2024). Intracolonial genetic variability, arising from mechanisms such as somatic mutations, polyploidy, and chimerism, provides a hidden reservoir of genetic diversity that can enhance the evolutionary flexibility and adaptation of coral colonies, providing an overlooked pool of genetic diversity to support rapid adaptation to marine heat waves, compared with the much slower process of adaptation by intergenerational selection through sexual reproduction. Most coral population genetic studies identify and remove clonemates to avoid inflating allele‐frequency estimates. Yet finer clonal growth processes that shape genotype spread and interaction within and among colonies are rarely considered. As a result, intracolonial genetic variability (IGV), despite its importance, is often overlooked, potentially leading to an incomplete understanding of coral adaptability (Re et al. 2024; Richards et al. 2023). Here, we argue that overlooking IGV may lead to an underestimation of the total genetic variation present in coral populations. Although population genetic methods are designed to infer diversity from representative subsamples, accounting for intracolonial variability provides a more complete understanding of the sources and structure of genetic diversity in clonal coral populations, particularly in the context of adaptation to environmental change. Hence, we emphasize the necessity of integrating IGV into coral population studies to achieve a more accurate assessment of their adaptive capacity. We discuss how high‐resolution genomic methods have replaced earlier genetic tools, allowing researchers to identify clones and IGV within colonies while simultaneously resolving fine‐scale population connectivity among reefs. Additionally, we explore the mechanisms generating intracolonial variability, its implications for genetic diversity, and its critical role in enhancing coral resistance and resilience under climate change. A basic model is also proposed to estimate the potential number of adaptive genotypes within coral colonies. By addressing these gaps, we advocate for a paradigm shift in coral population genetics research that recognizes IGV as a pivotal factor. Incorporating IGV into coral research, conservation, and restoration frameworks is essential for fully accounting for the adaptive potential of coral reefs in projecting future coral states and designing robust conservation strategies. Without this understanding, conservation efforts risk being built on an incomplete foundation, limiting their effectiveness in supporting coral population resilience and long‐term survival.

## 2 IGV in Scleractinian Corals

Scleractinian corals are modular, colonial organisms formed from multiple asexually produced integrated polyps (Oren et al. 2001) conferring a growth advantage to attain large isometric size and volume while maintaining a minimalistic base component unit as well as increasing surface area for exposure to vital resources such as sunlight and nutrition sources (Conlan, Bay, et al. 2018), whereas different colony regions may have specialized functions. For example, established colony regions are generally the most fecund, whereas the growing regions are often sexually sterile and allocate resources to further clonal growth (Nozawa and Lin 2014). Different colony regions may also have a strong labor division by possessing disparate biochemical compositions and exhibiting many differentially expressed genes (Hemond et al. 2014; Conlan, Bay, et al. 2018). Such intracolonial functional variability may be facilitated by clonal integration mechanics and further reinforced through the selection and maintenance of intracolonial genetic variability (IGV) (Conlan, Humphrey, et al. 2018), suggesting complex genetic and physiological dynamics within coral colonies, potentially influencing their adaptability and resilience (Maier et al. 2012). IGV, which has been observed for a number of scleractinian coral species, may also have implications in the species' responses to environmental stress by providing a mechanism for rapid selection and adaptation (Schweinsberg et al. 2015; Oury et al. 2020).

Two primary mechanisms were suggested as sources of intracolonial genetic variability in corals: (a) chimerism, involving multiple genetic cell lines from distinct origins, commonly because of the fusion of young organisms, and (b) mosaicism, more prevalent, mainly resulting from somatic mutations that transform a uniform organism into a mosaic of genotypes within the colony. Although chimerism is rare because of stringent environmental, organismal conditions, and immune responses, mosaicism represents a more common phenomenon because of genetic changes within clonal and modular organisms (Schweinsberg et al. 2015). Modular organisms like colonial corals can propagate somatic mutations during asexual reproduction, leading to the accumulation of genetic changes within the colony. These mutations are inherited by newly budded polyps and by fragments that detach and establish daughter colonies. Consequently, IGV can progressively accumulate whenever the propagating tissue already contains multiple genotypes. This process can significantly enhance colony adaptability, although its full potential remains underexplored (Hiebert et al. 2021). Regardless of the mechanism involved, different regions of the same colony may harbor distinct genotypes and can generate genetically different gametes. Although the complete set of intracolonial genotypes cannot be transmitted intact because gametes are haploid, post‐embryonic mutations arising in long‐lived stem‐cell lineages that contribute to gametogenesis can be inherited, exporting novel allelic diversity to the next generation (DuBuc et al. 2020; Vasquez‐Kuntz et al. 2022; López‐Nandam et al. 2022). These considerations reinforce the need for a multi‐level selection framework that evaluates selection acting on polyps, whole colonies, and their sexually produced progeny (Schweinsberg et al. 2014).

## 3 Clones and Clonal Growth in Corals

**FIGURE 1:**
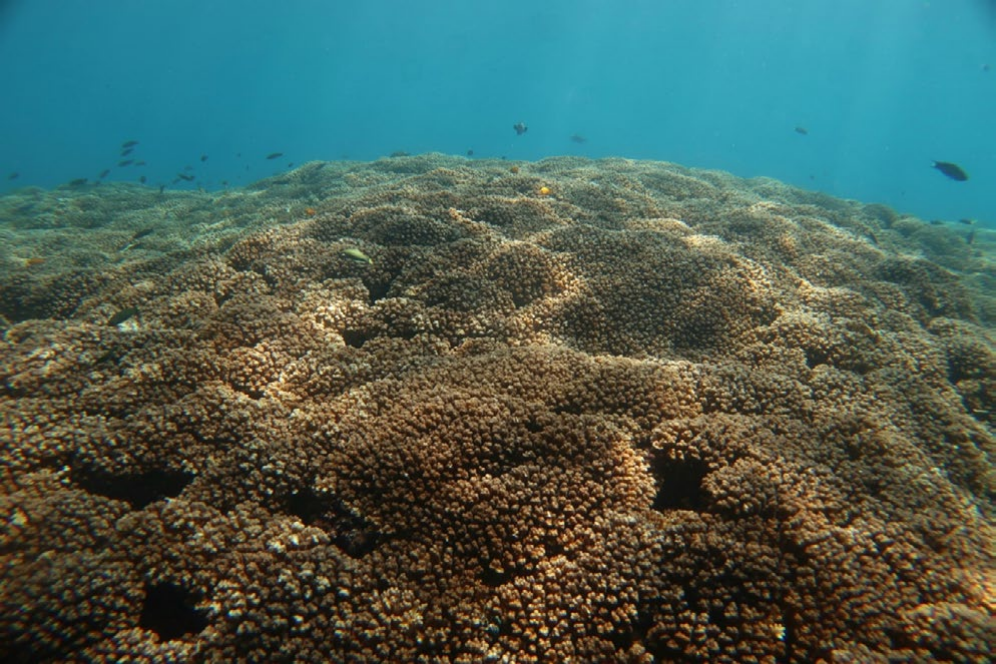
Large monoclonal stand of *Pocillopora acuta* at Terumbu Kili (5°43′53″ N, 102°59′52″ E) near Redang Island, Malaysia. Photograph: L. Afiq‐Rosli, April 2018.

More than 95% of all hexacoral species are colonial and exhibit clonal growth (Madin et al. 2016; Table S1, Data S1). This refers to asexual reproduction where new polyps are generated from existing ones, leading to the formation of genetically similar individuals within a colony and increasing the prevalence of clones (Baums et al. 2006; Pinzón et al. 2012). For example, a large extent of reefs can be made of a large monoclonal colony (Figure 1). Indeed, over 170 hexacoral species have a recorded maximum diameter of 1 m or more, with 30 of these exceeding 5 m in recorded maximum diameter (Madin et al. 2016; Table S2, Data S1). However, it is more likely that many smaller monoclonal colonies are formed when a large colony is fragmented by partial mortality, storms, wave action, or other mechanical forces (Highsmith 1982; Forsman et al. 2015; Capel et al. 2017; Caruso et al. 2021). When these colonies are sampled for assessing reef connectivity by elucidating population genetic differentiation in corals, clonemates are usually avoided. Sampling protocols often involve collecting colonies spaced at least 2 m apart to this end (Howlett et al. 2024), which have also been done with sourcing corals for experimental manipulations and coral restoration to avoid using similar clones (Baums et al. 2019; Afiq‐Rosli et al. 2019; Wee et al. 2019). Clonemates are also usually excluded from population genetic analyses (i.e., clone‐corrected) to prevent identical genotypes from skewing results and masking patterns of non‐random mating (Porto‐Hannes et al. 2015; Grünwald et al. 2017). As a result, only a handful of coral population genetic studies have to date evaluated intracolonial genetic variability in parallel with population genetic structure assessment (Schweinsberg et al. 2015; Oury et al. 2020; Oury and Magalon 2024). Consequently, the prevalent strategy for examining the population structure of corals usually involves focusing on just a single representative of each multilocus genotype. Therefore, there is a tendency to neglect intracolonial genetic diversity.

To better understand how this gap is reflected in the literature, we conducted structured searches on Scopus on the 21st of October 2024 using both broad and specific keyword sets. These included general terms such as “coral AND reefs” (35,589 results), targeted terms for genetic and genomic studies like “coral AND reefs AND genetics OR genomics” (2608 results), and highly focused terms for clonality and IGV such as “coral AND reefs AND clonality OR intracolonial variation” (147 results). Full details of the search queries, including seagrass‐related terms used for comparative purposes, are provided in Tables S3 and S4 (Data S1). Here, we found that when considering all coral reef research published in the peer‐reviewed literature since 1970, only 0.4% of studies have discussed intracolonial concepts such as clones, IGV, chimerism, mosaicism, and somatic mutations. Focusing specifically on coral genetics and genomics studies since 2000, we found that out of 2483 studies reviewed, only 5.93% addressed these intracolonial concepts. Early investigations of intracolonial variability and clonality in corals addressed four main questions. First, many studies used multilocus genotypes to infer the relative contributions of sexual recruitment and asexual fragmentation to population maintenance, especially in *Acropora* and *Porites* species (Ayre and Hughes 2004; Baums et al. 2013). Second, researchers examined fine‐scale population structure to test how clonal spreading influences patterns of gene flow and local relatedness (Combosch and Vollmer 2011; Foster et al. 2012; Devlin‐Durante and Baums 2017). Third, a growing body of work has linked intracolonial genotype mosaics to differential stress responses, asking whether certain ramets or genotypes within a colony perform better under thermal or acidification stress (Schweinsberg et al. 2015; Kitchen et al. 2020). Fourth, several experiments quantified fitness costs and benefits of chimerism or mosaicism by measuring growth, survival, and competitive ability of colonies containing multiple genotypes (Puill‐Stephan et al. 2012; Shefy et al. 2020).

**FIGURE 2:**
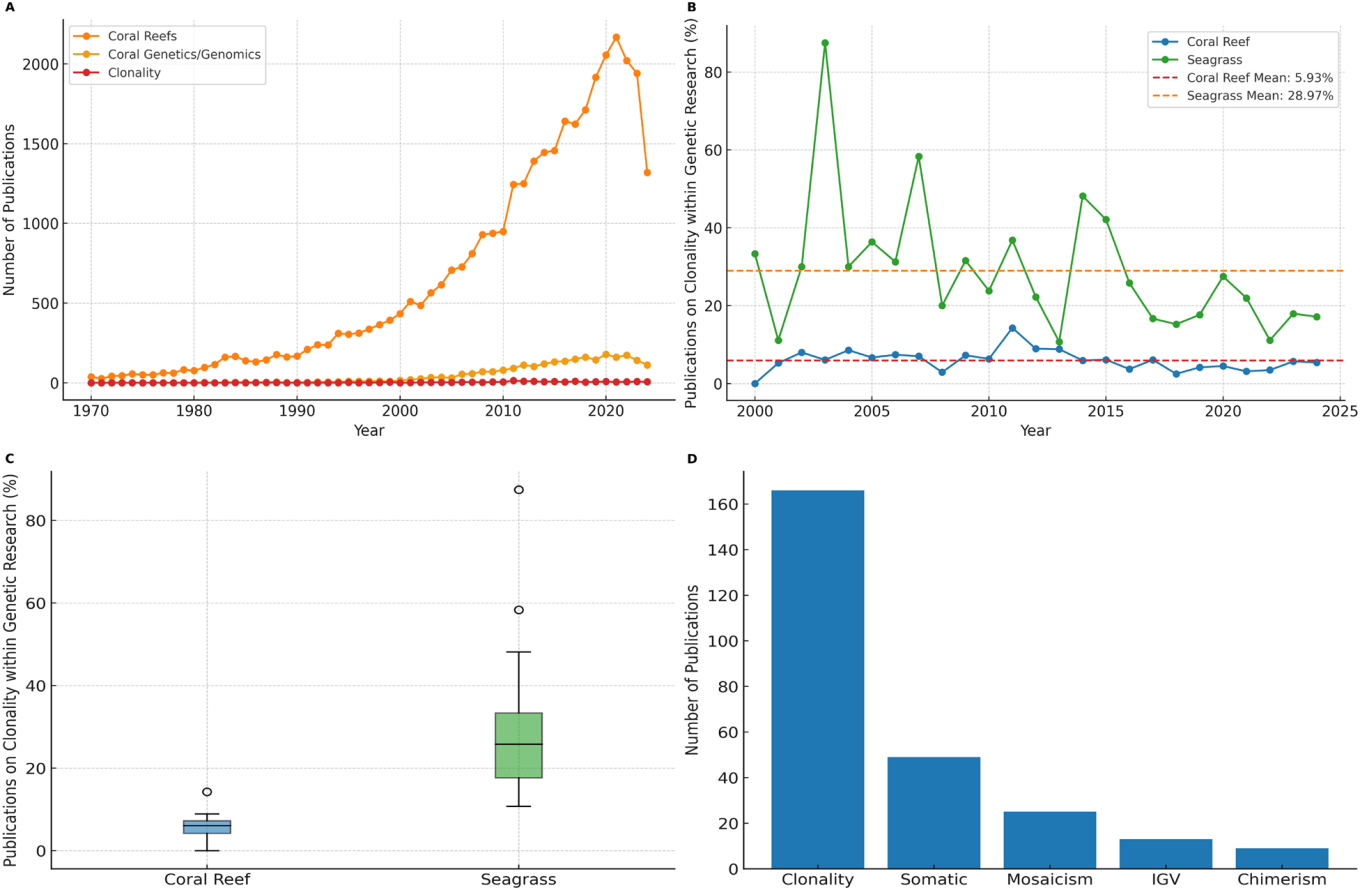
Overview of research publications related to coral reefs, genetics/genomics, and clonality. (A) Annual number of research publications related to coral reefs, coral genetics/genomics, and coral clonality from 1970 to 2024 (B) Percentage of publications focusing on clonality within genetic research for coral reefs and seagrass from the year 2000 to 2024 onwards. The solid lines represent the annual percentages. Dashed lines indicate the mean values over the period. (C) Distribution of percentages of publications referencing clonality within genetic research for coral reefs and seagrass from the year 2000 to 2024. (D) Distribution of publications mentioning clonality‐related concepts such as clonality, including ‘Clonality,’ ‘Somatic,’ ‘Mosaicism,’ ‘IGV,’ and ‘Chimerism. These studies were located through a search on Scopus and were then manually screened to remove unrelated articles. Refer to Tables S3 and S4 in Data S1 for a list of query terms used in the search.

By comparison, seagrass research, which is also strongly clonal, places much greater emphasis on these intracolonial concepts, with nearly 29% of seagrass genomics or genetics studies referencing intracolonial aspects (Figure 2). Seagrass research places a greater emphasis on clonal concepts because seagrasses often reproduce vegetatively, forming large, genetically identical patches that can persist for centuries (Rozenfeld et al. 2007; Arnaud‐Haond et al. 2007). This clonal growth is crucial for their survival and resilience, especially in environments where sexual reproduction is less successful. Understanding clonal dynamics, including intracolonial variation and somatic mutations, has been argued to be essential for assessing the genetic diversity and adaptability of seagrass meadows. This knowledge is vital for conservation efforts, as seagrass meadows play a key role in coastal ecosystems by providing services like carbon sequestration and habitat support (Procaccini et al. 2007; Waycott et al. 2009; Kendrick et al. 2019). The consideration of clonality in seagrass has generated a number of sampling, analytical, and computational approaches (Rozenfeld et al. 2007; Arnaud‐Haond et al. 2007, 2014; Arnaud‐Haond and Belkhir 2006; Bailleul et al. 2016), some of which have already been adapted for assessing clonality in corals. Given the critical role that clonal dynamics and intracolonial variation play in the resilience and adaptability of seagrass ecosystems and other modular organisms (see Hiebert et al. 2021), it is essential that coral reef research similarly integrates these concepts for a more accurate assessment of genetic diversity, which is required to inform conservation strategies that account for the true adaptive potential of coral reefs.

## 4 Methods to Detect Clones in Corals

A variety of techniques have been used to detect clones across diverse coral species and ecoregions worldwide, each offering unique advantages and limitations. (Table 1; Table S5, Data S1). The first method used to study clonality in marine life was histocompatibility tests, such as tissue grafting (Hildemann 1977). This technique was initially applied to identify whether branch segments in marine invertebrates, like sponges and cnidarians, were clonal (Jokiel et al. 1983; Neigel and Avise 1983). In this process, branches were tied together and monitored over a set period to check for tissue fusion, under the assumption that non‐clonal, histo‐incompatible tissues would not fuse and would exhibit necrosis. Subsequent research highlighted inconsistencies in this technique, casting doubt on this initial assumption, which was never independently verified.

**TABLE 1:** Strengths and limitations of various methods to detect clones in marine clonal organisms.

Method	Strengths	Limitation	References
Tissue grafting	Simple and direct physical method. Useful for studying histocompatibility and fusion in some marine organisms.	Limited to organisms where grafting is feasible. May not reliably identify all clones.	Hildemann (1977) Jokiel et al. (1983) Neigel and Avise (1983) Ayre and Willis (1988)
Gel‐based electrophoresis	Cheap. Simple to perform. Good for size‐based separation of DNA, RNA, or proteins.	Limited resolution compared to advanced DNA sequencing methods. Time‐consuming for a large sample size. Limited inference can be made for population genetics.	Ayre and Willis (1988) Coffroth et al. (1992) Sambrook and Russell (2001)
Amplicon sequencing	Cheap for a small sample size.	Not known as a method to detect clones. Limited resolution in population genetics.	
Microsatellites	High variability, reliable for population genetics. Robust statistics to detect clones. Minimal computational resources needed.	Labor‐intensive. Can be low resolution for the population genetics of certain species.	Liu et al. (2005), Arnaud‐Haond et al. (2007)
Reduced‐representation sequencing (e.g., RADSeq)	Provides a broad view of the genome. Good for population genetics. Robust statistics to detect clones.	Less comprehensive than full genome sequencing. Requires advanced bioinformatics for data analysis.	Locatelli and Drew (2019), Kitchen et al. (2020), Feldman et al. (2022), Oury and Magalon (2024)
Genome skimming/Low‐coverage whole‐genome sequencing (lcWGS)	Provides a comprehensive genome overview. Suitable for complex population genetic studies with a large sample size.	Lack of statistics to detect clones. Requires advanced bioinformatics for data analysis. Higher computational resources are needed.	Lou et al. (2021), Quattrini et al. (2023)
WGS	Provides a detailed genome analysis. Robust statistics to detect clones. Can identify somatic mutations.	Lack of statistics to detect clones in corals. Only suitable for a small sample size with current pricing. Requires advanced bioinformatics for data analysis. Higher computational resources are needed.	Reusch et al. (2021), Yu et al. (2024)

With advances in molecular techniques, allozyme electrophoresis emerged as one of the earliest methods for studying genetic variation in coral populations (Ridgway and Sampayo 2005). Although useful in detecting genetic variation, its resolution was limited, leading to its decline in favor of DNA‐based approaches (Allendorf 2017). Gel‐based methods, such as allozyme electrophoresis, remained a key tool for detecting clonal structures in early molecular studies (Willis and Ayre 1985). However, these approaches were time‐intensive and provided limited population genetic insights.

A breakthrough came with DNA fingerprinting, which revolutionized clone identification by using gel electrophoresis of DNA fragments to detect genetic differences more accurately (Coffroth et al. 1992). This technique allowed for a more detailed understanding of the clonal structure within marine invertebrate populations, surpassing previous methods in resolution and reliability. As sequencing technology advanced, microsatellites replaced DNA fingerprinting as the dominant tool for studying clonality, providing even finer‐scale genetic resolution.

Microsatellites, or simple sequence repeats (SSRs), provide a higher‐resolution insight into population structure and gene flow as well as improved clone detection. Used extensively in coral population genetics, microsatellites involve (1) estimating the probability that identical multilocus genotypes (MLGs) arise from separate zygotes and (2) analyzing pairwise differences among MLGs to detect somatic mutations or scoring errors (Liu et al. 2005; Arnaud‐Haond et al. 2007). Microsatellites remain widely used, with over 300 markers developed for 30 scleractinian species, half of which were designed in the last 10 years (See Table S6, Data S1 for a complete list). Although the detection of IGV depends on the number and polymorphism of loci examined, this method still offers a standardized framework for clone identification, enabling consistent comparisons across studies.

With advancements in molecular techniques, reduced‐representation sequencing (e.g., RADSeq and hybrid capture) has become a widely utilized approach for detecting clones and analyzing coral population structure (Combosch and Vollmer 2015; Oury et al. 2020; Bongaerts et al. 2021; Afiq‐Rosli et al. 2021, 2024; Buitrago‐López et al. 2023). This method generates a large number of single‐nucleotide polymorphisms (SNPs), making it a cost‐effective and scalable alternative to microsatellites. Additionally, it provides a broad genomic perspective and leverages robust statistical frameworks for accurate clone identification (Locatelli and Drew 2019; Kitchen et al. 2020; Feldman et al. 2022; Oury and Magalon 2024). Although microsatellites remain valuable, their labor‐intensive and species‐specific nature has led to an increasing preference for these high‐throughput genomic approaches. However, reduced‐representation sequencing requires advanced bioinformatics expertise and, unlike whole‐genome sequencing (WGS), does not provide comprehensive genomic coverage.

Building on this, low‐coverage whole‐genome sequencing (lcWGS) offers a broader genomic perspective and is particularly useful for studying population genetics in large sample sizes (Lou et al. 2021). However, its application in clonal detection is still limited because of the novelty of statistical methods required for accurate identification. At the forefront of coral genomic research, WGS provides the most detailed genetic analysis, allowing for precise clone detection and the identification of somatic mutations (Reusch et al. 2021; Yu et al. 2024). Technological advances have led to a 4000‐fold reduction in sequencing costs since 2010, making WGS increasingly viable for coral research (Locatelli et al. 2024). For instance, a study generated a chromosome‐resolved genome assembly for *Acropora millepora* , obtaining WGSs for 237 phenotyped samples, marking a step toward predicting bleaching response from genomic data (Locatelli et al. 2024). This method, however, is still constrained by financial and computational demands, particularly when applied to clonal organisms at scale.

## 5 Accumulation of Somatic Mutations During Clonal Growth

With each new polyp generated, a plethora of somatic mutations may occur, contributing to genetic diversity at the intracolonial level. These mutations, though happening at the somatic level, contribute to the genetic mosaic that characterizes individual coral colonies (Dubé et al. 2017; Olsen et al. 2019; López‐Nandam et al. 2022). A single colony, comprising hundreds to tens of thousands of polyps, holds the potential to generate novel genotypes through the accumulation of somatic mutations over time. For example, Van Oppen et al. (2011) estimated 100 million somatic mutations accumulated from both the endosymbiotic algae and coral host in typical 30 cm colonies of a branching coral.

This estimate could be higher for larger colonies and in coral colonies at shallower depths because of increased cell division and exposure to elevated thermal regimes and UV radiation, which increase the frequency of somatic mutations (Courtial et al. 2017; Olsen et al. 2019). As such, depth and colony size could predict the amount of accumulated somatic mutation. In the coral reef pathogen *Vibrio shilonii* , a decrease in pH levels was found to reduce mutation rates. This suggests that more acidic conditions, potentially because of ocean acidification, might similarly affect mutation rates in corals, although this requires further exploration (Strauss et al. 2017). The growth rates of individual species and how dense the polyp aggregates within a species could also be factors that contribute to higher somatic mutations. At such a high number, these mutations can promote adaptation because fewer cells containing beneficial somatic mutations are needed to enhance the fitness of the organism (Majic et al. 2022). An important advantage of clonal species is that deleterious mutations remove only the polyp affected, not the colony, from the population. Thus, the potential fitness cost of somatic mutations is minimal in clonal organisms compared to that of non‐clonal organisms.

Mosaicism, driven by the propagation of somatic mutations, introduces significant genetic variability within a colony. The modular nature of colonial animals, including corals, allows for delayed aging and indefinite growth through the accumulation of somatic mutations over time. Genetic mosaicism enhances the colony's capacity for adaptation in response to environmental stressors and, hence, its overall fitness (Hiebert et al. 2021). Whereas the majority of mutations may be expected to be selectively neutral, colonies may accumulate mutations with adaptive value as they grow, providing a source of novel genotypes that can be passed to their offspring (Vasquez‐Kuntz et al. 2022). This transgenerational translation of genetic diversity could potentially augment the adaptive capacity of coral populations, providing resistance against environmental perturbations. This is particularly important as clonal growth enables relatively rapid regeneration of coral cover from surviving colonies following disturbance, such as coral bleaching, compared to recovery via the much slower larval recruitment process (Linares et al. 2011; Gilmour et al. 2013).

## 6 Somatic Polyploidy

Whole‐genome duplication may occur in some cells and tissues of an organism, which results in these cells possessing multiple sets of chromosomes, a condition referred to as somatic polyploidy. The rise in chromosome numbers in polyploid cells amplifies genetic diversity, which serves as a basis for further changes in gene expression, epigenetic patterns, gene networks, protein profiles, cell size, and stress responses, providing a rich substrate for natural selection to act upon, driving adaptation (Fox et al. 2020). Nonetheless, debate continues over the effects of somatic polyploidy on organismal performance, which has been argued to be harmful because of its association with reduced proliferation, impaired regeneration, compromised cell functionality, and elevated genetic instability (Selmecki et al. 2015; Corneillie et al. 2018). In contrast, somatic polyploidy has been proposed to enhance cell function through the acceleration of metabolic activities, protein synthesis, regenerative capabilities, and prevention of oncogenic transformation (Anatskaya and Vinogradov 2010; Peer et al. 2017). Genomic duplications might also be selectively neutral, with polyploid cells functioning collectively like the equivalent number of diploid cells (Øvrebø and Edgar 2018; Anatskaya and Vinogradov 2022). The widespread occurrence of somatic polyploidy in nature, reported across a wide range of multicellular life forms such as algae, mosses, lichens, vascular plants, invertebrates, and vertebrates, suggests that it likely plays a significant role in adaptation across various species, particularly in their ontogenesis (Anatskaya and Vinogradov 2022).

**FIGURE 3:**
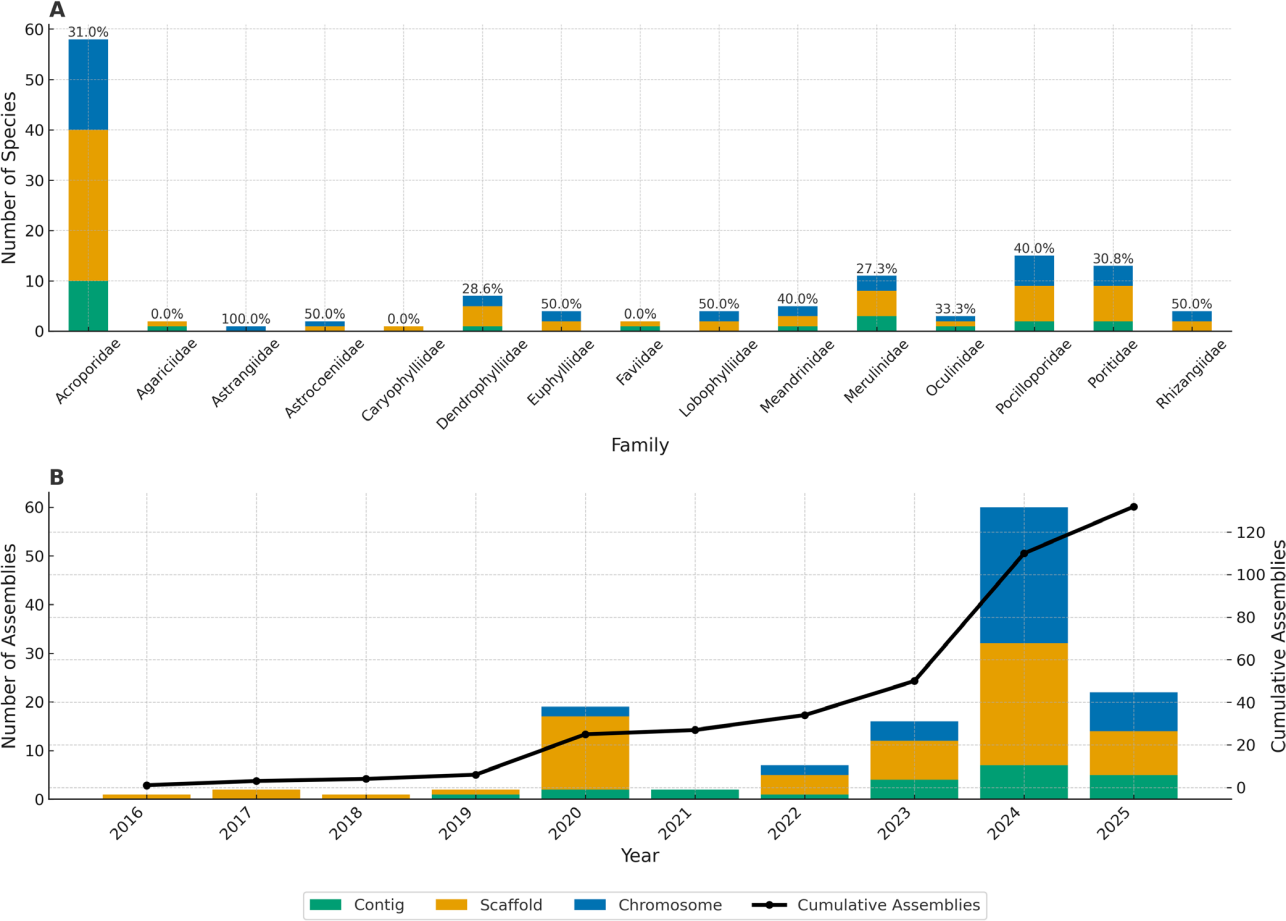
Publicly available genomes in scleractinian corals according to their families and assembly levels. Updated data from Pinsky et al. (2023) highlighting the percentage of chromosome‐level assembly per family. See Table S7, Data S1 for a full list of species.

For scleractinian corals, insights on polyploidy are still in their infancy but have been predicted to drive their evolution and adaptation (Kenyon 1997). However, with the lack of karyotypic data, this could only be examined in detail recently when chromosome‐resolved genome assembly data became available (Willis et al. 2006; Stephens et al. 2022). Indeed, a current search of the NCBI Datasets Genome database (1 June 2025) shows that 159 complete assemblies now exist for 79 species spanning 15 scleractinian families; Acroporidae alone represents about one third of these species, and although chromosome‐level assemblies first appeared only in 2020 and increased markedly in 2024–2025, they still make up fewer than one fifth of all assemblies, underscoring the need for additional chromosome‐scale genomes (Figure 3; Table S7, Data S1). Although these assemblies indicated that all sequenced genomes are diploid (Locatelli et al. 2024), with the exception of the genome of one species (*Pocillopora acuta*), the significance of polyploidy in scleractinian corals, including those in somatic cells, cannot be rejected. It was suggested that the evolution of polyploid genotypes in *P. acuta* (see Figure 1) may be explained by adaptation to local conditions, allowing them to outcompete ancestral diploid genotypes. Even though it is also more likely for polyploidy to arise from non‐somatic means, that is, through fertilization by foreign sperm on the already self‐fertilized eggs, or through the contribution of two closely related sets of alleles by a diploid gamete, its adaptive advantage is yet to be determined (Stephens et al. 2023). This is further confounded when intracolonial genomic variability and intergenomic variability between closely related genets are unexplored, as it is possible for local adaptation to be driven by somatic means (Majic et al. 2022). We, therefore, conclude that the full extent of somatic polyploidy in scleractinian corals should be explored further as part of efforts to assess modes of intracolonial genetic variability.

## 7 Chimerism

Chimerism in corals is a crucial evolutionary mechanism that can enhance intracolonial genetic diversity, offering a strategic advantage for survival in changing environmental conditions. This process involves the formation of chimeric entities from two or more genetically distinct conspecifics, leading to a collective that may possess a wider array of genetic and phenotypic potentials. In *Acropora millepora* populations on the Great Barrier Reef, chimeras were found to comprise a significant portion of the population (Puill‐Stephan et al. 2009). Half of *Pocillopora* sp. colonies in the southwestern Indian Ocean exhibited intracolonial genetic variability, with a portion of these being identified as chimeras, indicating that this phenomenon is not just an experimental artifact but a natural occurrence in coral reefs (Oury et al. 2020). Chimerism has been observed in both scleractinians and octocorals of the genus Corallium through aggregated larval settlement, where a considerable proportion of larvae settled near each other and fused, resulting in chimeric individuals (Puill‐Stephan et al. 2012; Giordano and Bramanti 2021; Jiang et al. 2022). Certain corals, such as *Pocillopora damicornis* , release asexually derived chimeric larvae, which exhibit genetic complexity and contribute to chimerism without requiring fusion (Rinkevich et al. 2016). Although less frequent, inter‐specific chimerism has been observed in some coral species, such as *Montipora capitata* , where larvae from different species fuse (Work et al. 2011).

Chimeric entities might exhibit enhanced capacity to respond to environmental stressors, such as those induced by climate change (Rinkevich 2019). Chimeras may combine the most advantageous genetic traits of their constituent genotypes, potentially offering a composite phenotype better suited to surviving in fluctuating or stressful environments (Rinkevich 2019). Indeed, chimerism could enhance coral fitness by increasing survival probabilities and promoting diversity in phenotypic traits, which could be crucial for mitigating the impacts of anthropogenic climate change (Shefy et al. 2020). However, partner genotypes may compete for limited resources, histoincompatibility can provoke partial rejection, tissue loss, or colony fission, and parasitic genotypes may overgrow cooperative ones, reducing overall fitness (Rinkevich 2002; Puill‐Stephan et al. 2012). Chimerism is still the least studied source of intracolonial variability in corals (Figure 2), so a systematic assessment of both its benefits and costs is needed to clarify its adaptive role.

## 8 Estimating the Number of Potential Adaptive Genotypes

The ability of a coral population to adapt to future disturbances lies within their genome and epigenetic mechanisms, with genetic variation arising through recombination during reproductive events or replication during colony growth, whereas epigenetic modifications can regulate gene expression in response to environmental changes (Bay et al. 2017; Dixon et al. 2018; Liew et al. 2020; Pinsky et al. 2023). Under certain assumptions, estimates of adaptive genotypes could be made. Given an average coral genome size of 6 × 10^8^ bp (see Table S7, Data S1) and a somatic mutation rate of 1 × 10^−7^ (Van Oppen et al. 2011; López and Palumbi 2020), we can infer that each cell division results in around 60 new mutations. Studies on *Acropora* and *Orbicella* colonies that screened thousands of loci found no verified back‐mutation events (Olsen et al. 2019; López and Palumbi 2020), indicating that reverse mutations occur at rates several orders of magnitude lower than μ and can be considered negligible for our calculations. Assuming 100,000 cells in a polyp (estimated from the number of coral host cells/30 cm colony, surface area/30 cm, colony (Van Oppen et al. 2011) and polyps per area (Madin et al. 2016)), as one new polyp is generated, there will be 4 million mutations accumulated. We assume a 1% chance for these mutations to be partitioned together during polyp division and a threshold for identifying a new genotype on the basis of a single nucleotide variants (SNVs) difference of 1 × 10^−4^ (which is a conservative 0.01% difference when compared with species variation of ~1% and population differences of ~0.1%), to calculate that to produce a new genotype, 60,000 such mutations are necessary. Hence, the genotype of the 1000th polyp produced (60,000/60) could be considered a new genotype relative to its parental polyp. When we also consider the heterozygosity ratio of 1:2 (López and Palumbi 2020), we can deduce that by the 3000th polyp clonally produced, the genotype will have heterozygous alleles, enhancing its survival prospects (Table 2).

**TABLE 2:** Mathematical framework for calculating genotypic and heterozygosity threshold in clonally growing coral.

Parameter	Symbol	Value	Calculations
Genome size	*G*	6 × 10^8^ bp	Given (Data S1)
Mutation rate	*μ*	1 × 10^−7^	Given (López and Palumbi 2020)
Mutations per cell division	*M*_division_	*G* × *μ*	(6 × 10^8^) × (1 × 10^−7^) = 60
Cells per polyp	Cp	10^5^ cells	Given (Van Oppen et al. 2011)
Mutations per polyp formation	Mp	*M*_division_ × *C*_p_	60 × 10^5^ = 6 × 10^6^
SNV difference	*S*_dif_	1% (1 × 10^−2^)	Given
Mutations required for a new genotype	*M*_g_	*M*_g_ × *S*_dif_	6 × 10^6^ × 1 × 10^−2^ = 6 × 10^4^
Polyp threshold for a new genotype	*P*_gen_	*M*_g_ × *M*_division_^−1^	6 × 10^4^/60 = 1000
Heterozygosity ratio	*H*_r_	1:2	Given (López and Palumbi 2020)
Polyp threshold for heterozygosity	*P*_het_	*P*_gen_ × *H*_r_	3 × 1000 = 3000

**FIGURE 4:**
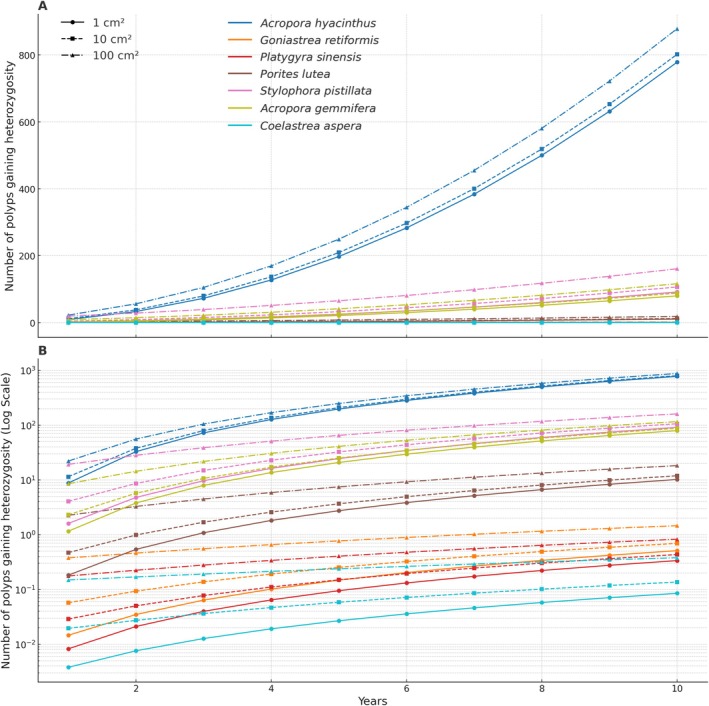
Number of polyps gaining heterozygosity over a period of 1 to 10 years for various coral species, starting from initial fragment sizes of 1 cm^2^, 10 cm^2^, and 100 cm^2^. (A) The data with a linear scale on the *y*‐axis. (B) The same data on a log scale, across a wide range of polyp counts. Each line represents a different species, with distinct markers and line styles used to indicate the initial fragment size. Coral species are selected on the basis of both growth rate and polyp/area data availability on Coral Traits Database (Madin et al. 2016; Table S8, Data S1).

The number of accumulated adaptive genotypes can, thus, be estimated on the basis of growth rates and the number of polyps per area of individual coral species. Using available data from the coral traits database (Madin et al. 2016; Table S8, Data S1), we predict that coral species with higher growth rates and polyp density, such as *Acropora hyachintus*, *Acropora gemmifera* , and *Stylophora pistillata* , should accumulate a higher proportion of adaptive genotypes compared to species with slower growth rates and lower polyp density, like *Goniastrea retiformis, Platygyra sinensis*, and *Coelastrea aspera* (Figure 4). This framework could be implemented in dynamic coral clonal growth models, such as that in Llabrés et al. (2024), which could simulate various genetic scenarios, thus providing a robust method to explore how clonal growth mechanisms contribute to genetic diversity and adaptation in coral populations. Although these estimates and calculations did not consider the complexity of various evolutionary processes and need to be tested by collecting and examining samples from the field, they can be considered as a first step toward modeling the adaptive capacity intraclonal genetic variability confers to coral species and the ecosystem they form.

## 9 Future Outlook and Recommendations

Here, we argue that intracolonial genetic diversity must have a large, but grossly overlooked, contribution to the adaptive potential of coral populations. There is, therefore, an urgent need for increased focus on the study of intracolonial genetic variability within coral populations, which is crucial for assessing the adaptive capacity of coral reefs to environmental stressors. Future research should prioritize methodologies that allow for the detailed examination of genetic variability at the intracolonial level, including advanced genomic techniques such as whole‐genome sequencing and reduced‐representation sequencing. The role of somatic mutations, polyploidy, and chimerism in contributing to genetic diversity and adaptability within coral colonies also warrants further investigation and, when sufficient empirical evidence arises, incorporation into models predicting future coral reef trajectories (Klein et al. 2024). Future population genetic and genomic research for scleractinian corals should consider employing a two‐pronged approach where the breadth of intracolonial genetic variability is examined in as many coral species and reef ecosystems as possible. Quantifying the extent of monoclonal colonies within these reefs is also crucial, as these potentially centennial‐old colonies face immediate threats from rapid ocean warming. These colonies could serve as critical resources for coral restoration efforts and as reservoirs of genetic adaptations essential for the resilience of future reefs. Initiatives like citizen science projects such as Map the Giants (Siena et al. 2024) should be supported and expanded to include not only massive coral species but also other coral growth forms. Novel remote sensing methods should also be developed to identify these colonies at scale.

By better understanding and harnessing intracolonial genetic diversity, scientists and conservationists can develop strategies that bolster coral resilience against climate change and uncover the adaptive mechanisms of corals. Recent work on coral restoration by Anthony et al. (2025) taps into the natural plasticity of the whole coral holobiont to boost resilience, an idea that matches the role of intracolonial genetic variability, also called IGV, as a source of adaptive potential. The method blends clonal fusion with microbiome tuning to raise colonies that can handle a wider range of stress. It relies on the agility and environmental memory of the corals' resident microbes, which pivot quickly when conditions turn harsh and help their hosts ride out the pressure. Grafting fragments that have grown in different settings brings the same benefit as IGV because a patchwork of genotypes widens the colony's functional toolkit and improves its odds of survival. Bringing the microbial and genetic angles together should give restoration stock more flexibility and a better chance of weathering the heat and acidity of a changing ocean. Overall, IGV looks less like an interesting quirk and more like a practical lever for assisted evolution and reef repair. This is crucial for crafting targeted, effective conservation interventions urgently needed to address the coral crises driven by steep climate change.

## Author Contributions

**Lutfi Afiq‐Rosli:** conceptualization (supporting), data curation (lead), formal analysis (lead), investigation (lead), methodology (lead), visualization (lead), writing – original draft (lead), writing – review and editing (equal). **Carlos M. Duarte:** conceptualization (lead), funding acquisition (lead), supervision (lead), writing – review and editing (equal).

## Conflicts of Interest

The authors declare no conflicts of interest.

## Supporting information


**Data S1:** ece372352‐sup‐0001‐DataS1.xlsx.

## Data Availability

Data used in this review is publicly available and have been included in a Supporting Information.
